# Development of a screening checklist to identify individuals with suspected allergy to polyethylene glycol

**DOI:** 10.1186/s44156-025-00100-4

**Published:** 2025-12-29

**Authors:** Jordan B. Strom, Nicholas Spetko, Yang Song, Constance E. M. Angell-James, Madeline A. Cassidy, Jessica L. Stout, Morgan L. Winburn, Rebecca Lee, Cosby A. Stone

**Affiliations:** 1 Division of Cardiology, Department of Medicine, Beth Israel Deaconess Medical Center, 375 Longwood Avenue, 4th Floor, Boston, MA 02215 USA; 2 Harvard Medical School, Boston, MA USA; 3 Richard A. and Susan F. Smith Center for Outcomes Research in Cardiology, Boston, MA USA; 4 Division of Infectious Diseases, Department of Medicine, Vanderbilt University Medical Center, Nashville, TN USA; 5 Division of Allergy, Pulmonary, and Critical Care Medicine, Department of Medicine, Vanderbilt University Medical Center, Nashville, TN USA

**Keywords:** Polyethelene glycol, Ultrasound enhancing agents, Safety, Allergy

## Abstract

**Background:**

Polyethelene glycol (PEG) is a key component of several ultrasound enhancing agents (UEA) but has been recognized as contributing to anaphylactoid reactions, resulting in new contraindications to use in those with known or suspected PEG allergy. Despite these recommendations, no clinical tools currently exist to screen for those with suspected PEG-allergy in echocardiography laboratories.

**Methods:**

We developed a screening survey to identify patients with potential PEG allergy and prospectively implemented it in a pilot study involving 8 patients with confirmed PEG allergy by skin prick testing and 50 prospectively enrolled patients undergoing clinically-indicated echocardiography without known PEG allergy, June – July 2025.

**Results:**

All patients completed the survey. A positive response to at least 2 of the first 4 questions on the screening survey had a sensitivity of 100% (95% CI 67.6–100%), specificity of 100% (95% CI 92.9–100%), positive predictive value of 100% (95% CI 67.6–100%), and a negative predictive value of 100% (95% CI 92.9–100%) to identify individuals with known PEG allergy.

**Conclusions:**

In this pilot multicenter study, a brief screening survey identified all patients with proven allergy to PEG, suggesting possible utility to its use to identify those with potential PEG allergy who would benefit from a non-PEGylated UEA, though further clinical validation is needed.

Polyethylene glycol (PEG) is a key component of several liposomal echocardiographic enhancing agents (UEAs), helping to stabilize the lipid shell and reduce microbubble opsonization and inter-cellular interactions [1]. At the same time, PEG, particularly high-molecular weight PEG, has been recently recognized as a key contributor to anaphylactoid reactions observed in individuals receiving such agents [1, 2], resulting in changes in labeling to include new contraindications to use of PEG-containing UEAs in those with known or suspected PEG-hypersensitivity [3]. While overall, the serious adverse event rate with receipt of UEAs remains low (~ 1:10,000) in contemporary practice [4], identifying the small number with suspected PEG-allergy represents a challenge given the ubiquity of PEG in thousands of enteral and parenteral drugs, many cosmetics and household products, and certain vaccines like the mRNA COVID-19 vaccines, with up to 24% of the population having been exposed to PEG [5]. As such, some individuals with PEG-allergy could be inadvertently administered PEGylated UEAs if prior allergic reactions are not recognized as being due to PEG.

As such, in this study, we sought to develop and prospectively validate a screening survey that could be administered to patients awaiting echocardiograms to identify those possibly requiring a non-PEGylated UEA. A checklist (Fig. 1) to screen for PEG allergy was developed in consultation with allergists with expertise in PEG allergy at Vanderbilt University Medical Center, Nashville, TN, USA (VUMC; RL, CAS) and echocardiographers with expertise in UEAs at Beth Israel Deaconess Medical Center, Boston, MA, USA (BIDMC; JBS, NS, JLS, MW).

**Fig. 1:**
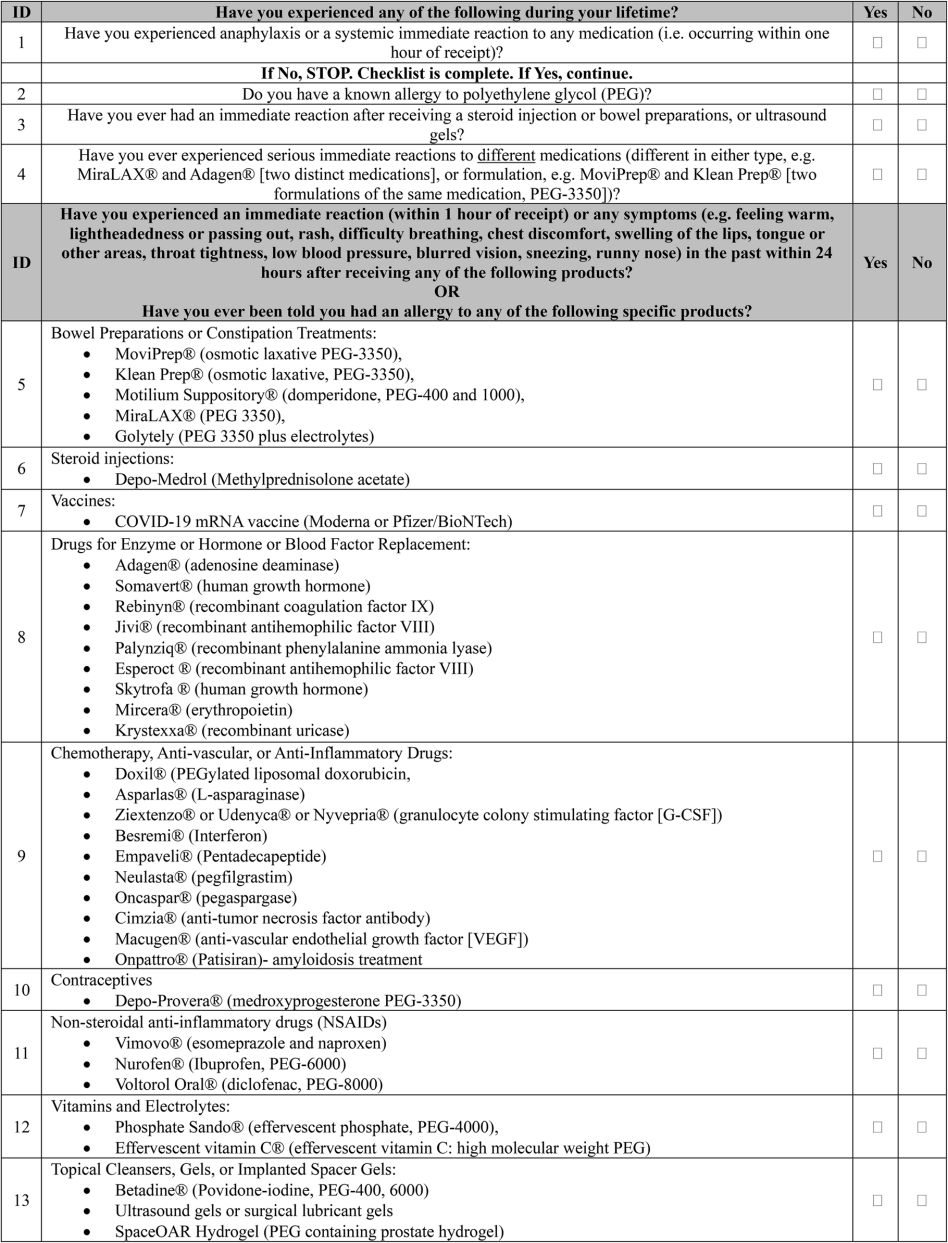
Survey questions to screen patients for allergy to polyethlene glycol. LEGEND: Shown are the survey questions administered to patients in the study to screen for allergy to polyethlene glycol (PEG). Questions are listed in order by identification (ID) number. For specific medications or agents, both brand name and generic name are provided with the list of agents included derived from prior publication (Stone CA, Liu Y, Relling MV, Krantz MS, Pratt AL, Abreo A, et al. Immediate Hypersensitivity to Polyethylene Glycols and Polysorbates: More Common Than We Have Recognized. *J Allergy Clin Immunol Pract.* 2019;7(5):1533–1540). Numbers following PEG (e.g. PEG-4000) represent the molecular weight with higher numbers indicating a greater number of ethylene glycol repeats

Delayed adverse cutaneous reactions to household products and cosmetics containing PEG were not considered as felt to occur via a different pathophysiologic pathway to PEG-mediated reactions to UEAs (which is felt to be either IgE-mediated or related to complement activation related pseudoallergy [CARPA]) [6]. This checklist was prospectively administered by front desk staff to 50 consecutive patients undergoing echocardiography at BIDMC and 8 patients with confirmed PEG allergy at VUMC [7] via telephone call and responses recorded, including one patient with a previous anaphylactoid reaction to PEGylated UEA [2]. No clinical details or identifying information was collected. The data collected from this study are available from the authors upon request.

A total of 58 individuals were administered the checklist between June 16, 2025 and July 3, 2025. All patients completed the survey and none declined participation. As written, those who answer “No” to the first survey question were instructed not to continue completing the survey. All except one patient at BIDMC answered “No” to question 1 with this patient responding “No” to questions 2–4. Of the eight PEG-allergic patients, 6 (75%) responded “Yes” to question 4 and all eight (100%) responded “Yes” to questions 1–3. A positive response to question 1 had a sensitivity of 100% (95% CI 67.6–100%), specificity of 94.0% (95% CI 83.8–79.7%), positive predictive value (PPV) of 72.7% (95% CI 43.4–90.3%), and negative predictive value (NPV) of 100% (95% CI 92.4–100%). A positive response to at least 2 of the first 4 questions had a sensitivity of 100% (95% CI 67.6–100%), specificity of 100% (95% CI 92.9–100%), PPV of 100% (95% CI 67.6–100%), and a NPV of 100% (95% CI 92.9–100%) to identify PEG allergy.

In this case-control study, a positive response to at least 2 of the first 4 questions on a screening survey identified all individuals with confirmed PEG allergy and none of those without PEG allergy. As PEG is present in two of three commercially available UEAs [1], either as an excipient (Lumason/Sonovue^®^, Bracco Diagnostics, Milan, Italy) or integrated into the microbubble shell (Definity^®^, Lantheus Medical Imaging, Billerica, MA), use of this survey in the waiting room could be used to screen patients presenting for echocardiography or contrast-enhanced radiologic ultrasounds who benefit from a non-PEGylated agent. Similar safety screens are utilized to evaluate for imaging-related risk in radiologic settings such as magnetic resonance imaging and computed tomography [3]. As no clinical information was acquired other than the questions in the survey, it is not possible to evaluate how this survey works across subgroups of interest. Moreover, as this represents a pilot study, further prospective clinical validation is needed. However, as UEAs remain underutilized in echocardiography [8, 9], administration of this survey could help promote broader adoption and safe use of this vital technology.

## Data Availability

The data collected from this study are available from the authors upon request.

## Abbreviations

BIDMC: Beth Israel Deaconess Medical Center
CARPA: Complement Activation Related Pseudoallergy
NPV: Negative Predictive Value
PEG: Polyethylene Glycol
PPV: Positive Predictive Value
UEA: Ultrasound Enhancing Agent
VUMC: Vanderbilt University Medical Center
